# Chemistry Central Journal themed issue: Current Topics in Chemical Crystallography

**DOI:** 10.1186/s13065-014-0069-9

**Published:** 2014-12-15

**Authors:** Mike Hursthouse

**Affiliations:** 1 Chemistry, Faculty of Natural and Life Sciences, University of Southampton, Southampton, SO17 1BJ UK; 2 Department of Chemistry, Faculty of Science, King Abdulaziz University, Jeddah, 21588 Saudi Arabia

## Abstract

No abstract

## Preamble

It was a pleasure to be invited to act as editor for this themed issue in which we aim to assemble a series of invited and submitted papers which provide a look at some of the current research efforts defining the subject of “Chemical Crystallography”. Of course, the timing of this is to make a positive contribution to the celebration of this special year, 2014 - the UNESCO Year of Crystallography.

I opted to use this generic title since I have always considered that it suitably described the kind of research in which I have been involved throughout my academic career – using crystallography to study chemistry! This view stems from the time I began my PhD, in the Chemistry Department of King’s College London. I joined a small group, supervised by Mr Ralph Hulme, and on my “recommended” reading list was the core text book entitled “Chemical Crystallography”, written by C.W.Bunn [1]. The first edition was published in 1945, and although it contained chapters on other facets of crystallography, a number of others did focus on topics of relevance to chemistry, and it provided many good links between the two areas. It is amusing to note that a Google search using this title does not yield a specific dictionary entry for the subject of “Chemical Crystallography” in Wikipedia, although many University “Chemical Crystallography Groups” are identified, and it conveniently links to the website of the Chemical Crystallography Group of the British Crystallographic Association, and through this, to many other National sites. The search also links to a site from which one can download a free copy of Charles Bunn’s book!

My PhD, which involved the preparation and structure determination of solid forms, which we called “packing complexes”, prepared by crystallizing antimony trichloride from some organic molecules, such as dibenzyl and stilbene, and for which I actually built my own low-temperature device for use with a Weissenberg camera with a split cylindrical cassette, was followed by a period as a postdoctoral research fellow with Professor Donald Rogers, a trained physicist with a Chair in the Chemistry Department at Imperial College, and a remarkable scientist and teacher. My area of research here was the structure determination of natural products, all solved by making heavy atom derivatives, but I can recall having my first experience with structure-solving by direct methods, using triple products to attempt to solve a structure in two dimensions - by hand! Before the completion of my Fellowship, I was fortunate to be invited to take up a position as Lecturer in Inorganic Chemistry at Queen Mary College London, where my brief was to assemble suitable equipment in order to apply X-ray crystallography to structurally characterise new kinds of compounds which were then being synthesised in inorganic chemistry.

Thus, in these early days, my main interests in Chemical Crystallography, like many of my cohort, were drawn into “molecular” science – discovering the distribution of atoms and groups in new molecules and complexes. On the one hand was the type of work I was involved with at Imperial College – the determination of the structures of organic natural products [2]. Even using film methods, a structure determination by X-ray crystallography was much quicker than several years of work based on systematic degradation and re-assembly. On the other hand was the determination of the structures of the new types of compounds which were being synthesised. In the beginning, the main emphasis was directed at metal-containing compounds, especially in the buzz area of “organometallic” chemistry, in which these compounds, with metal-to-carbon bonds were yielding new types of structures with new types of bonding and new types of reactions [3]. A related class of compounds, generally referred to as metal-organics, comprised structures with organic ligands, but with direct bonds from the metal to non-carbon atoms of the organic ligands, also opened up new channels [4]. The bonding in these types of compound often required new thinking and this created pressure to generate bonding interpretations of structure. Fortunately, new ideas and software on the description of chemical bonding were now also rapidly developing in theoretical chemistry, and the champions of the two approaches which could be applied to bonding in molecular structures - valence bond theory [5] and molecular orbital theory [6] - competed to provide models, which could also be tested using other, mainly spectroscopic techniques. In the same period, we were also seeing the first activities in the development of computer programs using the empirical methods of molecular modelling [7]. As in crystallography, the development of these topics benefitted enormously through the increasing power of computers. Indeed, a diversity of procedures and associated software were rapidly developed and integrated in the general area we now know as “Computational Chemistry”. This provides us with a richness of valuable tools, not only to work in molecular and solid state science, but in most areas and techniques used in chemistry.

## Advances in chemical crystallography

As our ability to determine and understand chemical structures developed, with the increasing numbers and power of automated X-ray diffractometers and the rate at which we were able to determine crystal structures, chemical crystallographers began to consider just how much information we could obtain from a structure determination. Instead of taking crystals from our colleagues just for structural characterisation, we began to take a broader look at the results we were accumulating. Less blinkered approaches than the classic interpretation of just the molecular chemistry led us to think more about the forces which held the crystal structures together, and thus more about the impact of these on the overall structures and properties of the solid state. From this point, a number of new themes and sub-topics began to emerge, which contributed to the widening scope of the subject. We consider some of these in the next sections.

### Intermolecular bonding and supramolecular chemistry

One of the first themes which emerged from our increasing interest in intermolecular bonding was a strong focus on hydrogen bonding strengths and patterns and, before very long, about variations in such patterns. This was not new science, with much of the ideas and groundwork having been laid for some time. The early work of Pauling [8], Powell [9] and Wells [10] and others, prompted the realisation that hydrogen bonding was not just a way of holding protein chains together [11], but a force to be recognised in chemical crystallography in general. The informative writings of Hamilton and Ibers [12], Jeffrey [13] and the papers of Etter [14],[15] were fundamental in promoting this subject, which provided most significant contributions to key areas such as the study of polymorphism [16] and, of course, the whole area of organic solid forms. These were the starting points for the conception of supramolecular chemistry [17] and the development of crystal engineering [18]. Advances in this theme have been spectacular, with many reviews, textbooks and Meetings. A flavour for what is currently in vogue is nicely summarised in the program of a recent Gordon Conference on the subject [19].

In a not unrelated way, studies on the synthesis and structural characterisation of metal co-ordination compounds, especially the use of bi- or multi-coordinating ligands led to the equally significant, and popular area of metal-organic frameworks, or MOFs [20]. In many ways, these types of compound have provided remarkable analogues of the structural characteristics and properties of zeolite and related phases, which had already been utilised as scaffolds for separation science and synthesis and catalysis [21]. Ian Williams and co-workers from Hong Kong University of Science and Technology: HKUST will be contributing to this Issue, with a paper on reduced symmetry of sodalite (SOD) MOFs and the concept of conformational isomers for these frameworks.

### Integrating computational chemistry and chemical crystallography

Knowing that the electrons in the molecule were responsible for the scattering of X-rays, the 1980’s saw experiments devised and performed to see whether we could determine and model not only the atomic positions and thermal motion characteristics, but the actual distribution of electrons, on the atoms and in the bonds. Initial work in this area, which became described as “Charge Density Studies”, and its development is nicely presented in a review co-authored by Philip Coppens, one of the early proponents of the subject [22], and taken up by many other researchers. The early work was based on data collected at low temperature on serial diffractometers, sometimes with supporting data from neutron scattering experiments. As in all other areas of x-ray scattering, major developments were made as the technology and data processing software for area detectors provided efficient collection of data of excellent quality, and yet another major theme grew. Integrating the experimental methods with new procedures for refining, which provided descriptions of the electron density in bonds, including intermolecular connections again led to a major involvement with computational chemistry methods. A number of recent publications highlight the impressive levels to which this area has developed, including the combined use of X-ray and neutron scattering to measure both charge and spin densities [23]-[25]. It is very pleasing to have an update on application of charge density studies in crystal engineering, from Piero Macchi and Anna Krawczuk, for this issue.

The rapidly increasing knowledge base on the properties and descriptions of interatomic and intermolecular bonding, coupled with the hugely increasing amount of structural data, fuelled another successful Chemical Crystallography/Computational Chemistry integration, in the form of Crystal Structure Prediction. New computational platforms were designed and assembled, which generated large numbers of possible structures and then classified them in terms of computed lattice energies. The remarkable success of this development is nicely charted in the reports of a series of competitive “Blind Tests” [26]-[31], which will continue through 2014/5. A very useful advantage of the computational procedures associated with this topic is the possibility to compute lattice energies for polymorphs in particular, and we have such an example in one of the “home” contributions to this issue, through a collaboration with Frank Leusen and John Kendrick from Bradford. Another contributed paper, from Thomas Gelbrich and Ulrich Griesser from the University of Innsbruck, will highlight the use of a further energy calculation program, PIXEL, written by Angelo Gavezzotti [32],[33], and will describe how the complete set of pairwise intermolecular interactions in a structure, from van der Waals to hydrogen bonds, can be computed, and how these interact in producing the final, overall energy.

### Crystallography under extreme conditions and other specialised experiments

Whilst variable, low and high temperature crystallography has become a very general technique, allowing detailed studies of phase transformations, for example, more specialised experiments involving studies under very high pressures have also yielded some very interesting results, particularly in the area, again, of phase transitions. The origins and development of the technique, the use of which is still not as widespread as low temperature crystallography, are nicely summarised by Andrzej Katrusiak [34]. Simon Parsons from Edinburgh and co-workers will be contributing a paper on a new study to this issue.

A second type of specialised technique, which has blossomed with the availability of pulsed synchrotron sources, is that of time-resolved crystallography. Here we irradiate a sample with pulsed radiation of a relevant wavelength and capture the diffraction pattern in synchonisation with the pulsing. Much of the work in this area is devoted to the study of macromolecules, but reports of studies on short-lived species in small molecule systems are increasing significantly. A review, summarising the history and the state-of-the-art in this technique has recently been published [35].

### Crystallographic computing, data bases and descriptors

Here we come to the crux of how we are really driving Chemical Crystallography, how we are storing our results and how we are using them. The first component of our toolkit are, of course the software tools used to drive the diffractometers, process the captured X-rays, solve and refine the structures and then display and interpret the results. I have experienced this development from the very beginning! My first structure was determined using eye-measured film intensities and calculation of 2D electron density maps using Beevers-Lipson strips! Fortunately this “good for the experience” procedure was then superseded by use of an electron density synthesis program, written by Owen Mills from the University of Manchester, and a least squares refinement program written by John Rollett at the University of Oxford. Diagrams were prepared using rulers, compasses and Indian ink. In contrast to this, the software we now have, for calculations and graphics, is state-of-the art, and we have all been very fortunate to have some superbly skilled crystallographic software experts to provide us with such facilities [36]. However, the problems we are now tackling continue to present new demands, so the development of the software continues apace. Richard Cooper, Amber Thomson and Pascal Parois have kindly agreed to contribute a paper to this Issue, describing strategies for handling bigger and bigger structures, and the way in which these are being implemented in the Oxford CRYSTALS package.

As a result of these highly successful developments, we are, of course living with a continuing data explosion. It goes without saying that this data can be really valuable, if we learn how to use it well, and we must have a way of preserving it, not just for posterity, but for persistent use and re-use. Of course, we are very fortunate to have groups of experts in crystallography and data base protocols, who are taking great care of this task also. The resulting databases cover all fields, and are all listed in the IUCr website [37]. The entries in the CSD currently total well over 700,000, and in all databases, well over one million. We visit the databases according to our areas of interest and activity, firstly, perhaps to check that a proposed structure determination is not a duplication (although this is not necessarily a bad thing), and then to use any significant important information which we find as accompanying data when we write up the results of our study. Many publications use sets of data to present comparative studies. The value of this data is truly immense, but it would be even more valuable if the data from all the structures, which we know lie in filing cabinets or on local computer archives, can be made available for database capture. I see from interrogation of the Cambridge Structural Database (CSD) that more of us are now submitting single structure data as “Private Communications”, and this is truly helping to increase content and thus value. I believe that with just a small amount of collective effort, we can add structures which may not be written up in Journal form as Private Communications, and aim for a CSD total breaking the 1 M mark within 3 or 4 years. Let us try!

In many cases, we can mine data from a database to study multi-structure relationships, make valuable comparisons and learn more about general or specific trends. For this purpose, we have to recognise the real situation this presents us with – “here are more than one million answers: what are the questions”? By this, I mean that we must prepare our database searching questions in a most careful way. For example, a simple statistical probe, requiring an answer equivalent to “yes” or “no” may tell us very little. Accordingly, we must develop our expertise in generating descriptors with which we can encode our questions so that the answers are suitably partitioned. For instance, suppose we wish to study the tendencies of an organic compound containing a particular functional group to crystallise as a hydrate. A search which gives the simple answer x% yes, (100-x%) no, has very little use. We would need to encode into our question other factors – what other functional groups are present, and, for example, what positional relationship they have to the target group?

Careful definition of descriptors is also vital in database mining to explore such questions as - “is my molecule/structure similar to any other” or “how similar is my molecule/structure to another”. This has been a critical component in the structural systematics studies which have been the focus of research in my laboratory for several years [38], which has involved development of the XPac concept and software [39]. As a second “home” contribution, my co-workers David Hughes, Thomas Gelbrich, Terence Threlfall and I will be contributing a paper in which we propose some new thoughts on the way in which one can describe, and thus compare, hydrogen-bonded networks. We hope this will be of interest to many readers.

Indeed, I hope readers will find all the papers we are presenting in this Issue to be interesting and be inspired to submit additions to the collection!

Mike Hursthouse.

Southampton and Jeddah, 2014.
